# Determination of Contact Resistance of Thermal Interface Materials Used in Thermal Monitoring Systems of Electric Vehicle Charging Inlets

**DOI:** 10.3390/ma17133103

**Published:** 2024-06-25

**Authors:** Monika Pieszka-Łysoń, Paweł Rutkowski, Magdalena Kawalec, Dominik Kawalec

**Affiliations:** 1 Faculty of Foundry Engineering, AGH University of Krakow, Reymonta 23, 30-059 Krakow, Poland; kawalec@agh.edu.pl; 2 Aptiv Services Poland SA, Powstańców Wielkopolskich 13, 30-707 Krakow, Poland; dominik.kawalec@aptiv.com; 3 Faculty of Materials Science and Ceramics, AGH University of Krakow, Mickiewicza 30, 30-059 Krakow, Poland; pawel.rutkowski@agh.edu.pl

**Keywords:** thermal interface material, Laser Flash Analysis, thermal contact resistance, electric vehicle, charging inlet

## Abstract

The rapid growth of the electric vehicle (EV) market is observed. This is challenging from a materials point of view when it comes to the thermal monitoring systems of charging inlets, for which requirements are very restrictive. Because the thermal conductivity of the thermal interface material is usually measured, there is a significant research gap on the contact thermal resistance of novel materials used in the electric vehicle industry. Moreover, researchers mainly focus on electrically conductive materials, while for thermal monitoring systems, the most important requirement is a high dielectric breakdown voltage. In this paper, the thermal contact resistance of materials for EV applications was thoroughly analyzed. This study consisted of experimental measurements with the Laser Flash Analysis (LFA) method, as well as a theoretical analysis of thermal contact resistance. The main focus was on the extraction of contact and material thermal resistance. The obtained results have great potential to be used as input data for further numerical modeling of solutions that meet strict thermal accuracy requirements. Additionally, the chemical composition and internal structure were analyzed using scanning electron microscopy, to better describe the material.

## 1. Introduction

Utilizing an electric drive in the electric automotive sector requires large energy storage and transmission. Due to unpredictable factors that can occur during the charging of electric vehicles, such as fluctuating electrical resistance due to aging or improper use, continuous temperature monitoring becomes inevitable [1]. The efficiency of temperature detection influences the electric vehicle charging process [1,2].

To achieve precise temperature detection during EV charging, three main conditions must be met. First, the system must exhibit low thermal resistance, *R_total_*, determining the thermal heat flux from the heating conductor to the sensor [2]. Second, a low heat capacity is essential, in order to avoid significant time delays between the sensor temperature and the actual connection temperature. Lastly, a high level of accuracy from the temperature sensor is important. Evaluating the efficiency of such a system may be performed by thermal numerical analysis, and thermal resistance is essential as a pivotal input parameter that must be known and incorporated.

**Figure 1:**
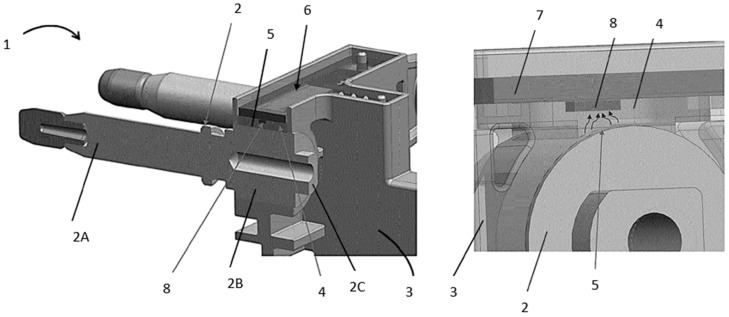
Example of a DC thermal monitoring system—1; with TIM—4; between the terminal—2, 2A, 2B, 2C, 5; and the sensor—8. Additional parts: housings—3, 6; PCB—7; patented by one of the authors of this article [3].

Thermal monitoring systems can utilize different material groups and various approaches to attach to the heating conductors. Parameters that need to be analyzed include the use of thermal interface materials (TIM) [3,4,5], thermal springs with TIM [3,6,7], and thermally conductive silicone rubber [8]. Each solution has different parameters that need to be analyzed. However, all materials used must have a high dielectric strength property value, because the most important requirement from the system’s point of view is protection against voltage breakdown between high-voltage and low-voltage lines. An example of the application is presented in Figure 1 in the patent application of one of the authors of this article [3]. The TIM, marked on the picture as 4, is the material under investigation in this work.

**Figure 2:**
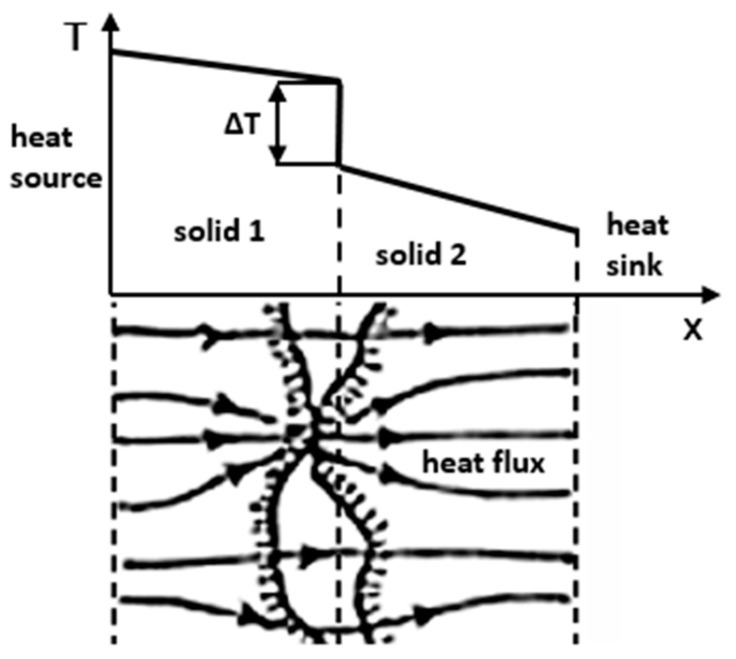
Temperature drop at the interface with the influence of surface roughness, own study based on [14,15].

In the case of TIMs, the most important parameter is thermal resistance, depending on the TIM thickness, usually described as Bond Line Thickness (BLT), and thermal conductivity and thermal contact resistance. Usually, TIMs are used as a thin layer of interface between a heat source and a heat sink [9] to replace low conductive air gaps and minimize the thermal contact resistance presented in Figure 2. The described work focuses on a TIM used as a dielectric layer with a thickness of up to 2–3 mm, placed between a heating conductor and a sensor of an electric vehicle thermal monitoring system. The efficiency of such systems is dependent on thermal resistance, which depends on the cross-sectional area of the conductive paste and thickness. Although the thermal conductivity of a material is usually provided in the manufacturer’s technical data sheets (TDS) [10,11,12], there is a significant research gap in the measurement of thermal contact resistance. The data is very limited because the measurement procedure is very sensitive in any setting and sample preparation, so it is no small task to conduct such an experiment. This type of material, used in the electric vehicle industry, is relatively novel and requires new solutions and analysis of thermal resistance [13].

The thermal contact resistance, $R_{contact}$, is described as a ratio between the drop of temperature, and the total heat flux over the interface [16]:

(1) $$R_{contact}=\frac{∆T}{q}$$

Contact thermal resistance causes a drop in temperature at the interface (Figure 2) since the surface roughness causes a decrease in the actual contact area between two surfaces compared to the apparent contact area [16]—Equation (1). The resistance depends on a contact area, which is determined by the surface roughness and the level of replacement of the air gaps by the thermal paste, which depends on the particle size (Figure 2). The existence of air gaps is undesirable because air has very low thermal conductivity ~0.026 W/(m∙K) [17] compared to the TIM 1–5 W/(m∙K) [9].

Evaluations of contact thermal resistance for TIMs and thermal pads can be performed by various methods, including stationary or transient, contact or non-contact, and destructive or nondestructive methods [18]. Swamy et al. [19], as well as Liu [20], introduced the utilization of the American Society for Testing and Materials (ASTM) D-5470 for TIMs for low-melting alloys, such as alloy/paraffin/olefin blocks. These methods, in comparison to LFA, do not allow for single layer measurements, or measurements of the sample in the air, without considering the contact thermal resistance. In the LFA method, there is also no thermal resistance at the heat source/sample and sample/probe boundaries. Hasselstrom [16] described a method to estimate thermal conductance for bolted joints, which is a very specific method and can only be repeated for metallic materials in certain configurations. Szałapak [21] and Zhao [22] used the LFA measurements for polytetrafluoroethylene materials and 3D graphene foam/polymer composites. Their research focuses on different types of materials and their analysis was more focused on the different shapes of the samples.

In this presented study, the LFA method was specifically chosen due to its non-contact nature and ability to measure small elements. This method enables the determination of the thermal diffusivity for a single-layered stand-alone sample, and also multi-layered samples (two- and three-layer methods) [23]. Furthermore, the LFA method facilitates precise thermal conductivity analysis across a wide range, from 0.2 to 400 W/(m∙K) [24], which covers the thermal properties of the materials examined (polymer and copper).

The main goal of this presented work was to determine the contact thermal resistance of TIM through the indirect measurement of the total thermal resistance and calculation of the theoretical resistance of the material. An additional aim was a comparison of thermal resistance determination methods, as well as the verification of the results on available data. All experiments were conducted using Laser Flash Analysis. Additionally, for better understanding of the material structure and behavior after sample preparation, Scanning Electron Microscopy (SEM) and Energy Dispersive Spectroscopy (EDS) analyses were performed. The data and results analyzed will be used in the heat transfer numerical models of the thermal monitoring systems of the charging inlets of electric vehicles. The properties of contact resistance for these materials have great influence from a thermal modeling standpoint. In the literature, an investigation of new materials combining SEM and thermal measurements was described by Lee et al. [25] and the combination of a numerical analysis with innovative materials analysis was presented by Yoo et al. [26].

## 2. Materials and Methods

### 2.1. Sample Description

**Figure 3:**
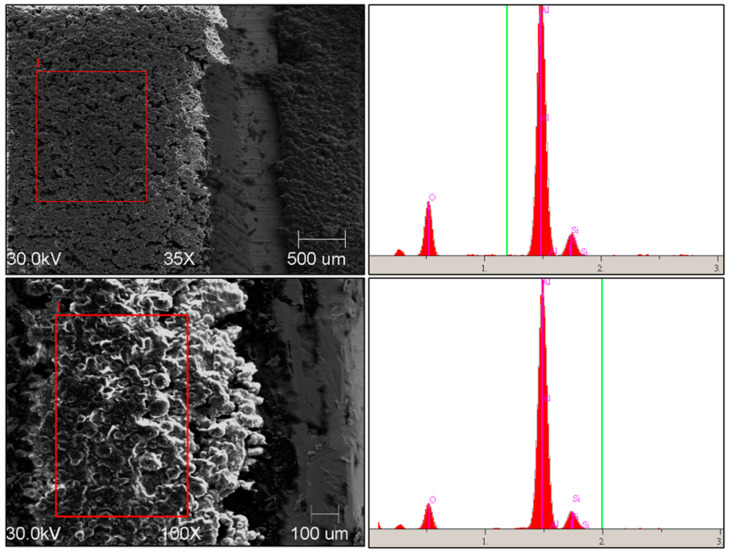
SEM image of material (**left**) and composition of the materials (**right**), measured for the 1 L sample (sample 1) and the 3 L sample (sample 4) from SEM-EDS; I—EDS analysis area.

Analyzed samples of thermal interface material (TIM) were made of thermally conductive silicon-based material with a pasty consistency, ensuring physical stability. It can be used as prefabricated thermal pads; a liquid gap filler, cured after dispensing (two-component materials); or a liquid not-curing gap filler (one-component material) [24]. Images of the material samples were made to observe the surface of the grains in terms of their shape. The research was carried out using SEM using a JEOL 5500 LV microscope with high resolving power and a Field Emission Gun (FEG) electron source. The topography of the sample was examined using semiconductor detectors. The beam energy was 30 keV. In selected areas, the chemical composition in microareas was analyzed using the EDS Ultim Max detector manufactured by Oxford Instruments. The chemical composition was analyzed as a map of the distribution of elements on the grain surface. Research was carried out on representative areas at a magnification of 35× and 100×. In Figure 3, which shows the composition of the material, mainly aluminum (Al), silicon (Si), and oxygen (O) are visible.

The main function of this type of material is to replace the air gaps between two interfaces with thermally conductive material [27]. TIM is characterized by a dielectric breakdown voltage at level 14 kV/mm and high thermal conductivity, *k* = 3.9–4.1 W/(m∙K) and 4.5 W/(m∙K). The properties of the material lead to the conclusion that this type of material would be suitable for efficient thermal monitoring systems with shapes that are difficult to fill with other materials, such as thermally conductive elastomers or thermal pads.

**Figure 4:**
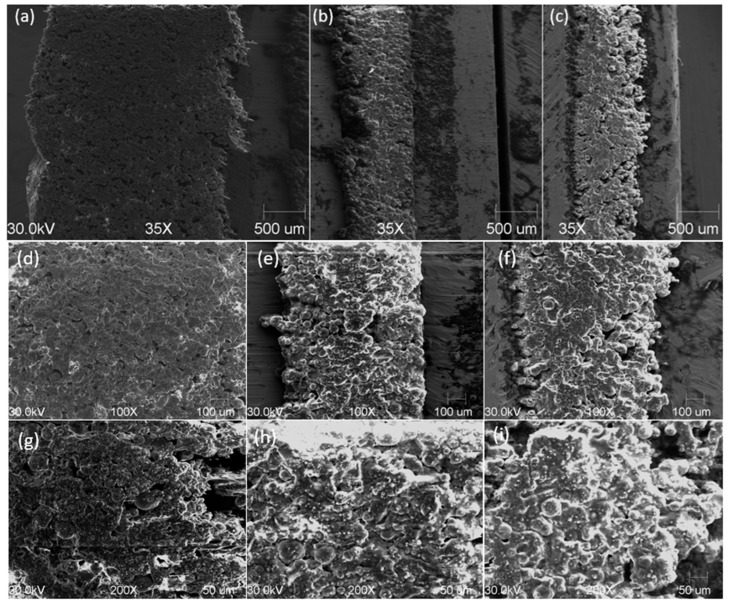
SEM images, magnification 35×: (**a**) 1 L sample 1, (**b**) 3 L 650 µm sample 5, (**c**) 3 L 100 µm sample 4. Magnification 100×: (**d**) 1 L sample 1, (**e**) 3 L 650 µm sample 5, (**f**) 3 L 100 µm sample 4. Magnification 200×: (**g**) 1 L sample 1, (**h**) 3 L 650 µm sample 5, (**i**) 3 L 100 µm sample 4.

In Figure 4, the SEM images of 3 samples of material 1 are presented: a single-layer sample (sample 1) and two three-layer samples of 650 µm (sample 5) and 100 µm (sample 4). No significant differences were noticed between the samples. The average grain size of the ceramic particles was 60 µm.

**Figure 5:**
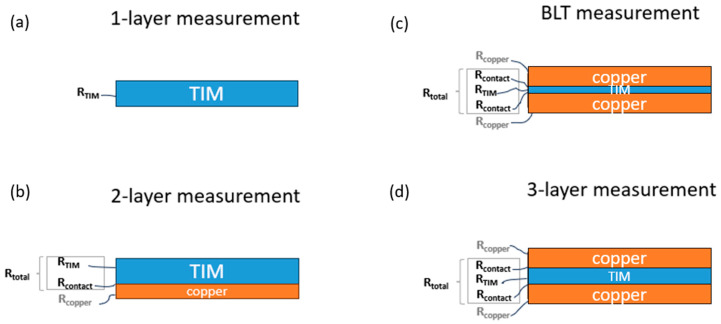
Different methods of measuring thermal resistance (**a**) single-layer material, (**b**) two-layer method, (**c**) BLT method, (**d**) three-layer method.

In this work, two different material types characterized by thermal conductivity of 3.9–4.1 W/(m·K) and 4.5 W/(m·K) were examined (Table 1). The name of the manufacturer was not provided, to indicate that no single material was preferred. The sample with 3.9–4.1 W/(m·K) was tested using the single-layer (Figure 5a), two-layer (Figure 5b), three-layer (Figure 5d), and Bond Line Thickness (BLT) (Figure 5c) methods, and 4.5 W/(m·K) samples were tested using only the BLT method (Figure 5c).

The following measurements were made as part of the research:

- Single-layer measurement

**Figure 6:**
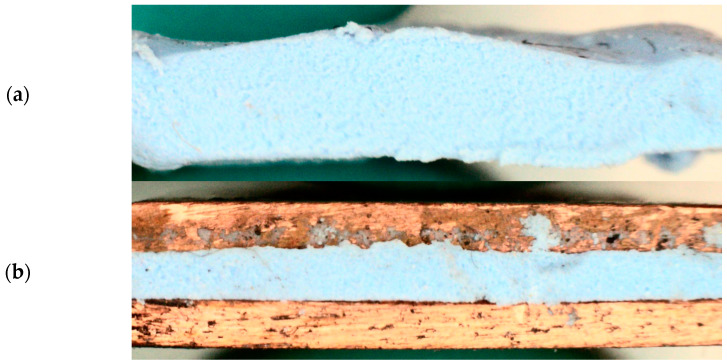
(**a**) Material before sample cut and cover with a graphite layer, (**b**) sample in three-layer and BLT configuration with copper plates on both sides.

A sample (sample 1) of 10 mm square and 1.66 mm thickness was prepared for a single-layer LFA measurement (Figure 6). The samples were coated with a Kontakt Chemie 33, Cramolin graphite layer on both sides to ensure a high level of emissivity (>95%) due to the use of an infrared detector (IR). Each sample was tested three times and the averaged value and uncertainty were determined (using Netzsch software Proteus LFA Analysis v. 4.8.5 and v. 8.0.3).

- Two-layer measurement

Two samples of 10 mm thickness square and 1.53 mm (sample 2) and 1.13 mm (sample 3) were prepared for two-layer LFA measurements. The samples were covered with a graphite layer on both sides. The first sample was tested once, the second was tested three times, and the mean value and uncertainty were determined.

- BLT measurement

A 3.9-4.1 W/(m⋅K) sample measurement and two 4.5 W/(m·K) measurements were prepared as follows: the TIM was placed between two copper plates with a 10 mm square shape. The copper plates were 0.8 mm thick and the BLT was 100 µm for the 3.9 W/(m·K) sample (sample 4) and 180 and 150 µm for the 4.5 W/(m·K) sample (samples 6 and 7).

- Three-layer measurement

A sample was prepared for three-layer LFA measurements (Figure 6) with a square shape of 10 mm and a thickness of 0.65 mm (sample 5). Each sample was placed between 0.8 mm thick copper plates and covered on the outside surfaces with graphite.

### 2.2. LFA Measurements

**Figure 7:**
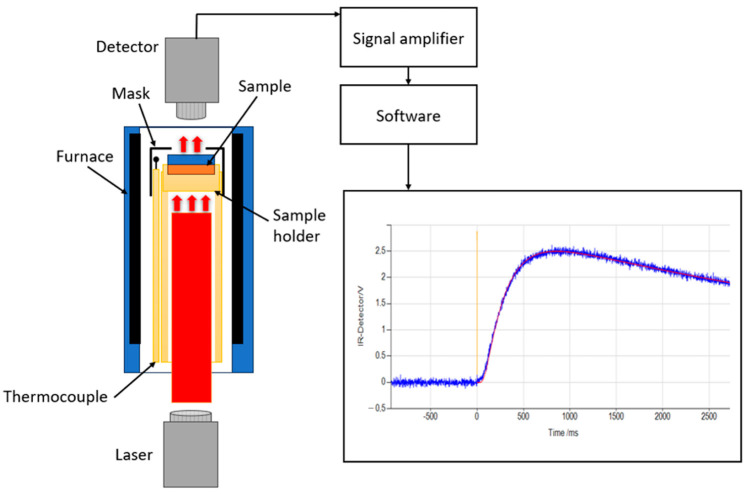
LFA devices with a scheme of the principle operation, based on [29,31,32].

LFA is a transient, non-contact method used to measure thermal diffusivity for a wide range of materials [28]. The tests in this work were carried out on Netzsch equipment (Selb, Germany) LFA 427, which is characterized by an uncertainty of 3% for thermal diffusivity, a range of 0.01 mm^2^/s to 1000 mm^2^/s, and high reproducibility throughout the application range from 20 °C to 2000 °C [29,30]. The principle operation of LFA is shown in Figure 7. The laser emitter generates a short laser pulse directed to the bottom side of the sample and its length is from 0.3 to 1.2 ms for LFA 427 [29]. The laser energy absorbed by the sample surface is up to 20 Joules per pulse, and the duration of the pulse is dependent on the sample type. The temperature of the upper surface is measured by an infrared detector over time, and the thermal diffusivity is calculated directly from the *t*_1/2_ coming from the curve (Figure 7) [22].

The measurements were carried out at an ambient 20 °C, in a protective atmosphere of argon or the air. The laser voltage was set to 550 V, a pulse width of 0.6 ms, and at the end, the pulse integral was 2.4188 and the amplitude gain was 127. During analysis, all parameters were selected based on multiple trials in order to obtain a proper pulse shape. Additionally, the influence of the protective atmosphere was checked and no changes in the results were observed. In the future, more advance studies on different ambient temperatures would be beneficial.

For all the single-layer samples, the Standard Cape-Lehman 1 L heat loss model was used [33]. For the two- and three-layer samples, the Lee model was used, where the thermal quadrupole method was applied. In the first method, the contact resistance is estimated simultaneously with the other parameters, including the thermal diffusivity of the unknown layer and the heat losses from the front and rear faces [33,34]. For the two-layer samples, the 2 L heat loss model with pulse correction + contact resistance was used in two options: known–unknown model and known–known. For the three-layer samples, a 3 L heat loss model with pulse correction as known–known was used.

Thermal diffusivity was measured in the single-, two- and three-layer systems using LFA. On the basis of this, the thermal conductivity and thermal resistance were determined. Lastly, using the BLT method, the total thermal resistance was measured and then the contact resistance was calculated.

### 2.3. Single-Layer Measurements

As a first step, measurements of a single-layer sample were performed.

**Figure 8:**
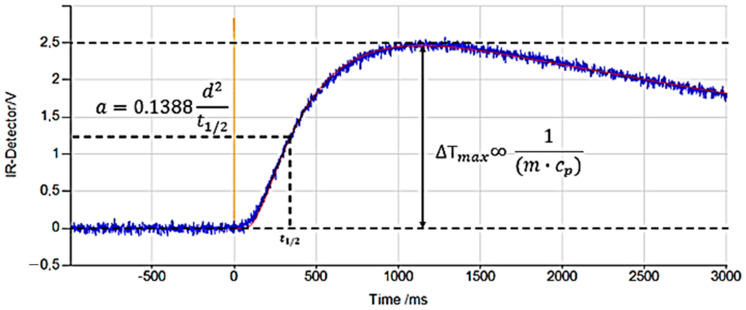
Representation of the *t*_1/2_ parameter based on the temperature changes detected by the LFA equipment from the moment of impulse start to stabilization. This example is from the two-layer measurement of sample 2.

In this step, the thermal diffusivity was a direct result and was calculated using the Formula (2) shown in Figure 8 [35]:

(2) $$a=0.1388\frac{d^{2}}{t_{1/2}}$$

Thermal conductivity was calculated using Formula (3), where diffusivity, specific heat, and density depend on temperature:

(3) $$k(T)=a\left(T\right)\cdot c_{p}(T)\cdot \rho (T)$$

Material thermal resistance $R_{TIM}$ (Figure 5a) is a function of thermal conductivity and the thickness of the sample:

(4) $$R_{TIM}=\frac{d_{TIM}}{k_{TIM}}$$

### 2.4. Two-Layer Measurement

In this method, a two-layered system was used, which consisted of a 0.8 mm plate copper and the TIM. The copper plate was introduced as a known material for LFA. Only the unknown diffusivity of the TIM was measured, and it consisted of *R_contact_*, which was part of the equation. *R_total_* is defined as *R_TIM_* + *R_contact_* (Figure 5b). Only *R_total_* was presented in the results.

### 2.5. BLT Measurement

In the BLT method, the sample is treated as a two-layer system composed of 2 copper plates as a known material, and the resistance (*R_total_*) is measured for the TIM layer in between. *R_total_* consists of *R_TIM_*, also known as BLT material thermal resistance, and two times *R_contact_* (Figure 5c). In the final results, the values of *R_contact_* were calculated based on the extrapolation of the graph curve and presented in the table.

### 2.6. Three-Layer Measurements

The three-layer system is proposed in this analysis. The laser beam was directed first to the copper plate and the direct result of thermal diffusivity contained a sample resistance and the contact resistance. Copper plates were not of interest in the analysis and were treated as known material and automatically subtracted by the Netzsch software LFA Analysis v. 8.0.3. Based on Equation (3), the effective thermal conductivity, which is a combination of the sample and contact resistance (Figure 5d), was calculated. The density and specific heat are given in the product data sheets. Using the electrical analogy, the effective thermal conductivity can also be presented as shown in Equations (5) and (6):

(5) $$k_{total=}\frac{d_{total}}{R_{contact}+\frac{d_{TIM}}{k_{TIM}}+R_{contact}}$$

The total thermal resistance of the system is defined in Equation (6):

(6) $$R_{total}=R_{contact}+\frac{d_{TIM}}{k_{TIM}}+R_{contact}$$

In the final equation, the resistances are considered as follows:

(7) $$R_{total}={2R}_{contact}+R_{TIM}$$

## 3. Results and Discussion

### 3.1. Single-Layer Measurements

At first, the thermal diffusivity for the single-layer measurement was determined based on a direct signal from the LFA experiment, and it was allowed to directly derive thermal conductivity and thermal resistance. Two tests were carried out at an ambient temperature of 20 °C.

Measurements conducted in the air and protective atmosphere of argon showed identical thermal diffusivity. The average value of the thermal diffusivity was 1.184 mm^2^/s and the maximum uncertainty of the three samples calculated in the software was ±0.003 mm^2^/s. After the calculations, the thermal material resistance of the TIM was equal to 4.9 (cm^2^∙K)/W and the thermal conductivity was equal to 3.39 W/(m∙K), which is a lower value than the 3.9 W/(m∙K) given in the technical data sheets. The reason for the discrepancy is likely that it is very difficult to maintain the proper thickness as a single-layer system for this type of paste-like material. For that reason, the two-layer system was further investigated.

### 3.2. Two-Layer Measurement

The thermal diffusivity for the two-layer measurements was determined based on the LFA direct result at an ambient temperature of 20 °C, and then the same method as on the single-layer system was used to derive thermal resistance and thermal conductivity, according to Equations (3) and (4). Later, the theoretical value of the resistance of the material was calculated based on data from the technical data sheet of the material, and contact resistance was obtained in the end—the results are presented in Table 2.

For sample 2. The thermal diffusivity of 1.347 mm^2^/s ± 0.009 mm^2^/s was measured, allowing a thermal resistance calculation of 4.019 (cm^2^·K)/W. For this sample, the contact resistance was equal to 0.096 (cm^2^·K)/W. For sample 3, the average value was calculated from three measurements, and the thermal diffusivity of 1.376 mm^2^/s ± 0.009 mm^2^/s was obtained, which gives the thermal resistance for sample 3 as 2.906 (cm^2^·K)/W, leading to an estimation of contact resistance at a level of 0.009 (cm^2^·K)/W. The results of contact resistance were compared with the literature 0.01–0.1 (cm^2^·K)/W [9] and the results obtained are in the defined range.

Furthermore, the thermal conductivity was calculated, which was 3.807 W/(m∙K) for sample 2 and 3.889 W/(m∙K) for sample 3. However, these values contain both the material and contact resistance.

### 3.3. BLT and Three-Layer Measurement

The third part of the measurement procedure was the analysis of the BLT system and the three-layer measurement. The physical construction of the sample was the same for both methods (Figure 5c,d). The difference was a definition of the Proteus LFA Analysis v. 4.8.5 and v. 8.0.3 and the output parameters. In the BLT method, the definition is provided as a two-layer system, and the output parameter is the thermal resistance. For a three-layer system, the definition of the system is the three-layer measurements and the thermal diffusivity in a direct output parameter. The BLT test may be performed when the layer is very thin, ensuring at least minor contact between solid materials [36]. For thicker samples, measurements have to be performed in a three-layer approach. For the materials examined, the BLT limit had been established at 200 µm [37], based on similar analyses available in the TIM technical data sheets. The total thermal resistance increased with the BLT value.

The direct result of the BLT method was the *R_total_*, which was 0.40 (cm^2^∙K)/W with BLT 100 µm for material 1, sample 4, and 0.47 and 0.55 (cm^2^∙K)/W with BLT 150 and 180 µm for material 2 samples 6 and 7. The uncertainty in this case was not given directly by Proteus LFA Analysis v. 4.8.5 and v. 8.0.3 and was considered to be the maximum equipment uncertainty, 3%.

The total thermal resistance was measured for sample 5 of material 1 indirectly using the LFA three-layer method and the thermal diffusivity of 1.677 mm^2^/s ±0.011 mm^2^/s. The *R_total_* was calculated using Equations (3) and (4) as 1.37 (cm^2^∙K)/W for a TIM layer thickness of 650 µm.

**Figure 9:**
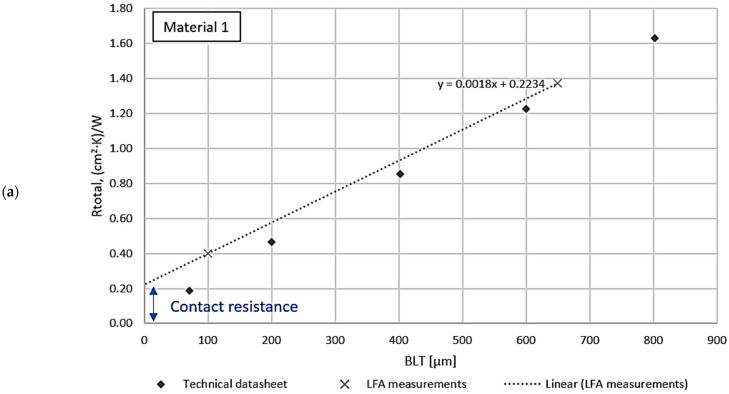

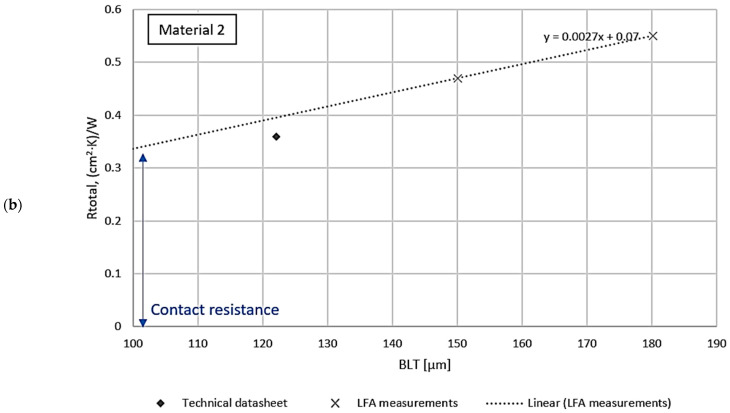
Verification of the results compared to the technical data sheet results (**a**) for material 1, (**b**) for material 2.

Due to the fact that, in reality, thermal conductivity may be slightly different from that defined in the TDS, this method is not the most accurate—Equation (7). The contact thermal resistance, presented in Table 3, was calculated by extrapolating the linear trendline from the measured data points (Figure 9), based on Equation (7), but without explicitly estimating *k_TIM_*. For material 1, thermal resistance was calculated as 0.1 (cm^2^⋅K)/W (half of 0.2 (cm^2^⋅K)/W from the graph—(Figure 9)) and 0.17 (cm^2^⋅K)/W (half of 0.34 (cm^2^⋅K)/W from the graph—(Figure 9)) for material 2.

The results were verified by comparison of the total thermal resistance with the example data sheet for both materials and presented in Figure 9. For material 1, a curve was given, and for material 2, only one point in the example TDS was given. Maximum discrepancies were on the level of 0.1 (cm^2^·K)/W for material 1 and 0.05 (cm^2^·K)/W for material 2. A very good fit of the results to the original data proves that further conclusions regarding the contact resistance value are also very close to reality.

The final results of the contact resistance, determined as 0.1 (cm^2^·K)/W and 0.17 (cm^2^·K)/W, were compared to the typical TIM known on the market. A value of 0.39 (cm^2^·K)/W was presented in the technical data sheet for gap filler [38]. For thermal pads [39,40,41], the contact resistance is higher and is in the range of 1.68–9.7 (cm^2^·K)/W.

The data collected play a key role as an input parameter for numerical analyses. Based on the thermal analyses performed, it was investigated that a small change of just 2 (cm^2^·K)/W (very common for TIM) in the definition of the contact thermal resistance value can change the performance of the thermal monitoring system by a factor of 2. For example, the performance, defined in this application as the temperature difference between the heating terminal and the sensor, may be estimated in the simulation as 3 K due to the lack of contact resistance data, when, in reality, it can be twice as high, which is beyond the required limit for this type of system (usually 3–4 K).

## 4. Conclusions

The investigation presented and discussed in this study focused on an innovative material dedicated to the thermal monitoring system of the electric vehicle charging inlet. The materials analyzed have not been fully examined so far and there is no data in the literature on thermal contact resistance for these types of materials. The lack of data is probably connected to the high precision requirements for sample preparation and a very demanding measurement procedure, which requires long-term searching for the best device parameter setting operations and the relaxation time applied to the silicon-based material (structural stability).

The composition of the material was examined using a scanning electron microscope and mainly aluminum, silicon, and oxygen were detected. The internal structures of the samples were examined and no significant differences were found between the single-layer and three-layer samples.

Measurements show that the two-layer, three-layer, and BLT methods give a rational level of results when it comes to thermal diffusivity and contact resistance (which do not exceed a value of 0.17 (cm^2^·K)/W). The single-layer method is a method used solely to estimate the thermal diffusivity of a material, but it has certain limitations since it is very difficult to maintain the shape of the material after curing without using additional plates to form it.The two-layer method allows for the calculation of contact resistance based on the measured total resistance and the theoretical value of material resistance, which may lead to inaccuracy if the source data are not correct. The BLT and three-layer methods allow for the estimation of contact resistance without the need to base it on theoretical material resistance. For better accuracy of the obtained data, further measurements may be conducted by adding the points to the curve to increase the accuracy of the function calculated on the basis of it.

The thermal resistance measurement data gathered play a pivotal role in enhancing the accuracy of numerical analysis for systems involving the analyzed material. Subsequent stages of this research will be introduced as input parameters for the thermal model and will be explained in further articles. This presented work showed the differences between the measurement methods in LFA and compared the thermal resistance between the methods and the referenced values. The presented research and collected data fill the gap in the TIM contact resistance properties, providing valuable resources for fellow researchers. The results and the proposed methodology offer a valuable tool to analyze the thermal resistance of various materials.

## Figures and Tables

**Table 1:** Properties of the materials measured from the example data sheet.

Source	Calculated from the Technical Data Sheet	Example Technical Data Sheet Data		
Parameter	Thermal Diffusivity, mm^2^/s	Thermal Conductivity, W/(m∙K)	Density, kg/m^3^	Specific Heat, J/(kg∙K)
Material 1	1.451	3.9–4.1	3140	900
Material 2	0.929	4.5	2900	1670

**Table 2:** Summary of the averaged results from two-layer LFA, marked directly measured data.

Measured Parameter	d_total_, mm	a_total_, mm^2^/s	k_total_, W/(m∙K)	R_total_, (cm^2^∙K)/W	R_TIM_, (cm^2^∙K)/W	R_contact_, (cm^2^∙K)/W
Sample 2	1.53	**1.347 ± 0.009**	3.807	4.019	3.723	0.096
Sample 3 (avg. from 3 measurements)	1.13	**1.376 ± 0.009**	3.889	2.906	2.897	0.009

**Table 3:** The thermal resistance of the BLT two-layer method, marked directly measured values.

Measured Parameter	BLT/d_total_, µm	a_total_, mm^2^/s	R_total_, (cm^2^∙K)/W	R_contact_, (cm^2^∙K)/W
Material 1 sample 4 (BLT method)	100	-	**0.40**	0.10
Material 1 sample 5 (three-layer method)	650	**1.677 ± 0.011**	1.37	
Material 2 sample 6 (BLT method)	150	-	**0.47**	0.17
Material 2 sample 7 (BLT method)	180	-	**0.55**	

## Data Availability

Data are contained within the article.

## Nomenclature

*R_contact_*	contact thermal resistance between the copper plate and the thermal interface material; (K⋅m^2^)/W
*R_TIM_*	material thermal resistance of the TIM, (K⋅m^2^)/W
$R_{total}$	total thermal resistance, (K⋅m^2^)/W
*T*	temperature, K
a	thermal diffusivity, m^2^/s
$a_{TIM}$	thermal diffusivity of the TIM sample, m^2^/s
$a_{total}$	thermal diffusivity of the whole system, m^2^/s
$c_{p}$	specific heat capacity, J/(kg∙K)
d	thickness of the sample, m
$d_{TIM}$	thickness of the TIM sample, m
$d_{total}$	total thickness of the sample, m
k	thermal conductivity, W/(m∙K)
$k_{total}$	thermal conductivity of the whole system, W/(m∙K)
$k_{TIM}$	thermal conductivity of the sample, W/(m∙K)
q	heat flux, W/m^2^
t_1/2_	half time, time to reach half the signal height, s
∆T	temperature difference, K
ρ	density, kg/m^3^
